# Association Between Consumption of Foods Classified by Chewiness Level and Overweight/Obesity Among Israeli Adolescents: Insights from a Cross-Sectional National Youth Survey

**DOI:** 10.3390/nu18213677

**Published:** 2026-11-06

**Authors:** Tzach Itzhak Gil, Tali Sinai, Chen Dor, Vered Kaufman-Shriqui

**Affiliations:** 1 Department of Nutrition Sciences, Faculty of Health Sciences, Ariel University, Ariel 40700, Israel; giltz@ariel.ac.il; 2 Division of Nutrition Unit, Sheba Medical Center, Ramat Gan 5262100, Israel; hen.hadad@mail.huji.ac.il; 3 Israel Center for Disease Control, Israel Ministry of Health, Ramat Gan 5262100, Israel; tali.sinai@moh.gov.il; 4 School of Nutritional Sciences, The Hebrew University of Jerusalem, Rehovot 7610001, Israel

**Keywords:** BMI, childhood obesity, mastication, overweight, pediatric

## Abstract

**Background/Objectives:** Food texture and oral processing characteristics may shape eating behavior, dietary intake, and body weight, yet the role of food chewiness in adolescent weight status remains underexplored. This study examined associations between intake across levels of food chewiness and weight status among adolescents in a national youth nutrition survey. **Methods:** This secondary cross-sectional analysis used data from the Israeli National Youth Health and Nutrition Survey (MABAT Youth Second Survey). Food Frequency Questionnaire (FFQ) items were classified by an expert panel into five chewiness levels, from 0 (lowest) to 4 (highest). Weight status was determined using body mass index (BMI) z-score categories. Multivariable survey logistic regression models examined associations between intake at each chewiness level (per 10-g/day higher reported intake) and overweight/obesity, adjusted for age, sex, population group, socioeconomic status, and total daily energy intake. Linear regression of the five fully adjusted log odds ratios on chewiness level assessed the trend, incorporating their full variance–covariance matrix estimated from 2000 school-level bootstrap resamples. Confidence intervals and two-sided *p*-values for the trend were calculated using approximate Wald t inference with 207 degrees of freedom. **Results:** The analysis included 3428 adolescents (mean age 15.2 ± 1.6 years; 47.7% male). Higher consumption of foods classified at greater chewiness levels was associated with higher odds of overweight/obesity, with the strongest associations observed for levels 3 and 4. After adjustment, each 10-g increase in intake at chewiness level 2 was associated with 0.2% higher odds of overweight/obesity, whereas each 10-g increase at levels 3 and 4 was associated with 0.7% and 0.6% higher odds, respectively. The estimated linear trend across chewiness levels was β = 0.00137 on the log odds ratio scale per one-level increase (95% CI: 0.00055–0.00220; two-sided Wald *p* = 0.0011). **Conclusions:** Higher reported intake of foods assigned to higher estimated chewiness levels was associated with greater odds of prevalent overweight/obesity after covariate adjustment. The cross-sectional design does not establish the temporal direction of these associations or a causal effect of food chewiness on weight status.

## 1. Introduction

Obesity during childhood and adolescence is associated with adverse health outcomes and an increased likelihood of adult obesity and chronic conditions such as type 2 diabetes (T2D), cardiovascular disease (CVD), and sleep apnea [1,2,3,4]. Understanding the relationship between food characteristics, dietary intake, and weight status is therefore relevant to nutritional research. Among the sensory characteristics of foods, texture may influence food acceptance and food choices. Preferences for particular textures can affect children’s willingness to consume different foods, while the physical properties of foods may influence how they are processed in the mouth [5,6,7,8,9].

Research has documented differences between foods in the number of chews, oral exposure time, and expected satiation [9]. Studies in children and adolescents have also examined associations between eating behaviors and weight status [10,11]. In children, a systematic review found more consistent associations between weight status and eating rate and bite characteristics than with chewing-specific measures, for which definitions and findings varied considerably [11]. Related research examining texture preferences and consumption of fiber-associated foods among children aged 9–12 years found no overall association between preferences for soft or hard foods and BMI-for-age percentiles [12]. These studies address behaviors during eating or preferences for particular textures. A distinct question is whether the amount habitually consumed from foods classified by their estimated chewing requirements is associated with adolescent overweight/obesity.

The present study investigates this dietary exposure question using an expert-based classification of food frequency questionnaire (FFQ) items into chewiness levels. Here, chewiness refers to the estimated chewing requirements assigned to foods by clinical dietitians. Applying this classification to reported dietary intake enables examination of consumption within each level in relation to measured weight status in a large adolescent sample. This approach provides a practical framework for an exploratory analysis of food consumption according to estimated texture-related characteristics. The classification requires validation against objective measures and does not directly assess participants’ eating rate, number of chews, or other aspects of masticatory behavior.

Accordingly, this cross-sectional study examined the association between consumption of foods classified by chewiness level and overweight/obesity among adolescents aged 12–18 years, using dietary intake and measured anthropometric data from the Israeli National Youth Health and Nutrition Survey (MABAT Youth Second Survey).

We hypothesized that patterns of consumption across food-chewiness levels would be associated with the likelihood of overweight or obesity among adolescents, independent of age, sex, population group, socioeconomic status (SES), and total daily energy intake.

## 2. Materials and Methods

### 2.1. Study Design and Sample

This study conducted a secondary data analysis of the Second Israeli National Youth Health and Nutrition Survey (MABAT Youth 2), a nationally representative, school-based cross-sectional survey conducted between December 2015 and March 2016 [13]. The study received approval from Ariel University’s Institutional Review Board (AU-HEA-VKS-20240407).

The original MABAT youth survey design and operation have been described in detail elsewhere [13]. Briefly, this was a cross-sectional, nationally representative, school-based survey that included 7th- to 12th-grade students. The survey questionnaires were self-administered and included demographic and health-related information, and food intake behaviors were assessed through a set of direct questions (providing internal construct validity). Anthropometric data were measured in person. The study received approval from the Israeli Center for Disease Control. The study was reviewed and approved by the Ethics Committee of Sheba Medical Center (SMC-1570-14) on 9 October 2015, and complied with the Ministry of Education’s requirements. Schools were randomly selected from the Israeli Ministry of Education’s school list, with students clustered within schools. School compliance was high (93%).

### 2.2. Sample Selection and Study Size

**Figure 1:**
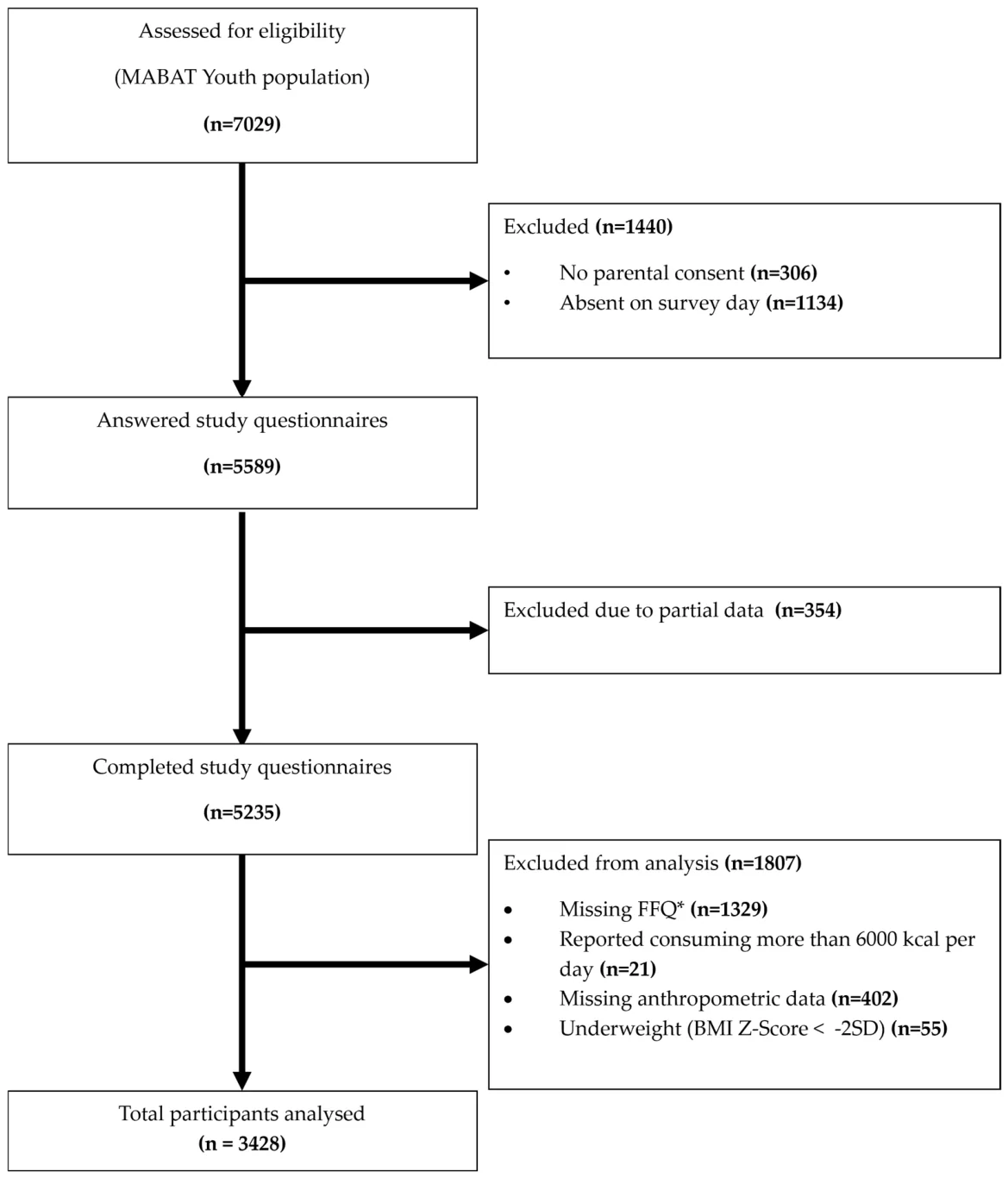
Flowchart of study participants. BMI, body mass index; FFQ, food frequency questionnaire; SD, standard deviation. * Missing FFQ also encompasses FFQs reporting an intake of less than 500 kcal per day.

The study population comprised participants in the MABAT Youth 2 Survey with usable FFQ data and measured height and weight. Of the 5589 respondents, 354 were excluded from the survey dataset due to incomplete data or insufficient response integrity. Among the remaining 5235 participants, exclusions for the present analysis were missing or ineligible FFQ data (*n* = 1329), reported energy intake above 6000 kcal/day (*n* = 21), missing anthropometric data (*n* = 402), and underweight status, defined as a BMI z-score below −2 SD (*n* = 55). Underweight participants were excluded because the analysis compared participants with normal weight with those with overweight/obesity. The final analytic sample included 3428 adolescents (65.5% of the 5235 participants retained after initial data quality screening; Figure 1). Included and excluded participants had similar mean ages (15.20 ± 1.59 vs. 15.06 ± 1.73 years, respectively). Compared with the excluded group, the analytic sample included a lower proportion of boys (47.7% vs. 56.8%) and higher proportions of Jewish adolescents (78.2% vs. 68.9%) and adolescents from medium-high socioeconomic backgrounds (67.3% vs. 49.2%).

The sample size was determined by the availability of eligible records in the existing survey dataset. All participants who met the analytical eligibility criteria were included; no separate a priori sample size calculation was performed for this secondary analysis. Illustrative sample-size calculations under alternative assumptions are provided in the Supplementary Table S1.

### 2.3. Weight Status Classification

Participants’ height and weight were measured by trained survey personnel using standardized equipment [13]. BMI z-scores were calculated and classified according to the WHO growth reference standards [14]. Weight status, the primary outcome, was categorized as either “normal weight” (BMI z-scores between −2 and <1 SD), “overweight” (1 ≤ BMI z-scores < 2 SD), or “obese” (BMI z-scores > 2 SD).

### 2.4. Chewing Level Categorization

The Chewability of foods was classified by a team of four clinical dietitians specializing in pediatric obesity, who classified the FFQ food items based on a literature review. Each dietitian independently rated food chewiness on a 0–4 scale, followed by group discussions to finalize classifications. Weighted averages were assigned to foods lacking consensus. The classification included: Category 0: Liquids (e.g., water, soft drinks, milk, dairy beverages); Category 1: Soft foods (e.g., yogurt, dairy desserts, ice cream, soft cheeses, spreads); Category 2: Moderately chewable foods (e.g., hard cheeses, cooked vegetables, baked cakes, breads); Category 3: Firm foods (e.g., fresh fruits, vegetables, meat products, packaged snacks); and Category 4: Hard foods (e.g., carrot, apple, cookies, hard-to-chew snacks) (Appendix A). Based on FFQ data, total food intake for each chewing level was calculated per participant. These independent variables were expressed as grams per day. The chewiness levels represent broad, expert-derived categories of estimated chewing requirements. Assignment to the same level does not imply equivalent nutritional composition, degree of processing, or identical physical properties. For example, nuts, cookies, apples, vegetable salads, and cereal bars were classified at Level 4 because the evaluators judged their chewing requirements to be relatively high within the proposed classification; this grouping was not based on quantitative measurements demonstrating equivalent chewing demands.

### 2.5. Additional Participants’ Characteristics

#### 2.5.1. Sociodemographic Characteristics

Age, sex, and school level (middle/high) were obtained from students’ responses. The population group (Jewish/Arab) was defined by the school’s language classification, according to the Israeli Ministry of Education (Hebrew/Arabic). SES was defined based on school-assessed welfare levels and categorized as low or medium-high SES, as previously reported [15].

#### 2.5.2. Health-Related Behaviors

Health-related behaviors were classified as previously documented [16]. Physical activity (type, frequency, and amount) was reported and classified as adherence to the recommended average duration of at least 1 h per day [17]. Sleep duration was also documented; a sleep duration of at least 8 h per day (for students aged 13–18 y/o) and 9 h per day (for students aged 11–12 y/o) was classified as recommended sleep [18]. Screen time was calculated by the time duration students reported watching movies/videos and using television, computers, tablets, and smartphones. Screen time was subsequently categorized according to adherence to the recommendation of no more than two hours per day [19,20].

#### 2.5.3. Nutritional Intake

Dietary intake was assessed using a 103-item FFQ. The FFQ was calibrated using dietary data from a subsample of 11.5% of survey participants who completed interviewer-administered 24-h dietary recalls. The calibrated FFQ data were used to estimate each participant’s habitual daily intake of the individual food items.

For each participant, the estimated daily intake in grams of all FFQ items assigned to the same chewiness level was summed to calculate total food intake at each of the five chewiness levels. These variables were expressed as grams per day. Total reported energy intake was calculated in kilocalories per day. Macronutrient intake was calculated in grams per day and expressed as a percentage of total energy intake.

#### 2.5.4. Statistical Analysis

Statistical analyses were performed using SAS version 9.4. The normality of the distributions of continuous variables was assessed using the Kolmogorov–Smirnov test. Normally distributed variables were described as Means and standard deviations (SDs). Variables with distributions significantly deviating from normality were reported as medians and interquartile ranges (IQR: 25th–75th). Categorical variables were presented as frequencies and percentages. These descriptive statistics were calculated using survey sampling weights (weighted by sex, primary language, and school level) to provide estimates representative of Israeli students in grades 7–12. Participants reporting energy intake >6000 kcal/day (approximately 3.5 SD above the sample mean) were excluded as extreme observations, consistent with a criterion previously applied in dietary research [21]. Differences in demographic, anthropometric, nutritional, and behavioral characteristics between the group with normal weight and the group with overweight or obesity were examined using the Mann–Whitney U test for continuous variables and the Chi-square test (χ^2^) for categorical variables. Multivariable survey logistic regression models were conducted to examine the associations between the amount of food consumed at each chewing level (unit: 10 g) and overweight/obesity, accounting for the sampling design and survey weights. Schools were specified as the primary sampling units for variance estimation to account for school-level clustering. Variables identified as significant in univariate analyses (*p* < 0.05) were entered into multivariate logistic regression models after testing for multicollinearity. Sex, age, and sociodemographic status were retained in all models as control variables. Models were first adjusted for age, sex, SES, and population group (Model 1); the second model included further adjustment for total daily energy intake (Model 2). Odds Ratios (ORs) and confidence intervals (95% CIs) are presented. All tests were two-tailed and considered significant at *p* < 0.05.

Linear regression was used to assess the trend across chewiness levels in the association between food intake and overweight/obesity. Because logistic regression coefficients (log odds ratios) possess approximately normal sampling distributions, the five fully adjusted odds ratios from Model 2 (per 10-g/day higher intake) were analyzed on a logarithmic scale. These log odds ratios were then regressed on chewiness levels coded from 0 to 4. To account for unequal precision and dependence between estimates derived from the same participants, the full variance–covariance matrix was estimated using 2000 joint Rao–Wu rescaled school-level bootstrap samples. In each sample, 207 schools were drawn with replacement from the 208 schools, all participants within the selected schools were retained, and survey weights were multiplied by the school selection multiplicity and 208/207. All five survey logistic regression models were refitted within each sample. The resulting full bootstrap variance–covariance matrix was incorporated into the regression estimation and standard-error calculations. Confidence intervals and two-sided *p*-values for the trend were calculated using approximate Wald t inference with 207 degrees of freedom (Supplementary Table S3).

## 3. Results

### 3.1. Study Sample and Participant Characteristics

In total, 2330 were classified with normal weight, 785 were classified with overweight, and 313 were classified with obesity. Compared to adolescents with normal weight, those classified with overweight or obesity were generally younger, with a higher proportion being male, a lower proportion being of Jewish background, and a lower percentage attending high school (Table 1).

### 3.2. Assessment of Inter-Rater Agreement in Food Chewiness Classification

Inter-rater agreement was evaluated using the four dietitians’ independent ratings recorded before group discussion; the final consensus classifications were excluded from this assessment. Ordinal Krippendorff’s alpha was calculated to account for the ordered five-level classification and the involvement of multiple evaluators [22]. The primary analysis included 99 of the 103 numbered FFQ items with four unambiguous numeric ratings. Four items were excluded because their entries contained preparation-dependent classifications, uncertain ratings, or alternative scores. A 95% percentile confidence interval was estimated using 10,000 bootstrap resamples of food items, retaining all four ratings together within each resampled item. Exact agreement among all four evaluators was also calculated. Ordinal Krippendorff’s alpha indicated high inter-rater agreement across the 99 FFQ items with four unambiguous numeric ratings (α = 0.943; 95% bootstrap CI: 0.901–0.971). All four dietitians assigned the same chewiness level to 77.8% of these items. The sensitivity analysis, including 102 items (excluding the carrot item, which remained excluded because its scores depended on preparation), yielded a similar estimate (α = 0.940; 95% CI: 0.898–0.968).

### 3.3. Food Intake Across Chewiness Levels and Associations with Overweight/Obesity

Food intake did not differ significantly between the weight-status groups at chewiness levels 0, 1, and 3. Adolescents with overweight/obesity reported a lower median intake of level 2 foods than adolescents with normal weight (Table 1). In Model 1, adjusted for age, sex, socioeconomic status, and population group, food intake at chewiness levels 0–3 was not significantly associated with overweight/obesity. In Model 1, a significant positive association with overweight/obesity was observed only for level 4 food intake. Following additional adjustment for total reported energy intake in Model 2, a positive association for chewiness level 2 approached statistical significance (*p* = 0.053), while statistically significant positive associations were observed for chewiness levels 3 and 4. Increments of 10 g/day in level 3 and level 4 food intake were associated with 0.7% and 0.6% higher odds of overweight/obesity, respectively (Table 2). No statistically significant associations were observed for levels 0 and 1. The adjusted odds ratios for overweight/obesity per 10-g/day higher food intake increased across chewiness levels (trend slope on the log odds ratio scale per one-level increase: 0.00137; 95% CI: 0.00055–0.00220; *p* for trend = 0.0011; Supplementary Table S3).

## 4. Discussion

Our findings indicate an association between the consumption of foods grouped using an expert-derived chewiness classification and adolescents’ weight status. Notably, greater intake of foods classified as having a higher level of chewiness (levels 3 and 4) was associated with increased odds of being overweight or obese, with a 10-g/day higher intake corresponding to 0.7% and 0.6% higher odds, respectively, after adjustment for total daily energy intake. This association should be interpreted cautiously because the cross-sectional design does not establish causality or temporal direction.

Although several associations were statistically significant, their magnitude per 10 g/day increase in intake was small, and their clinical relevance remains uncertain. The positive trend across chewiness levels indicates progressively larger log-odds coefficients, but does not establish a clinically meaningful effect. These estimates describe relative differences in the odds of prevalent overweight/obesity, rather than changes in body weight or equivalent percentage differences in outcome probability. Furthermore, the heterogeneous nutritional composition of foods within each chewiness category limits the translation of these associations into specific dietary recommendations. The findings therefore support further investigation of food chewiness within broader dietary patterns, but do not establish that modifying chewiness would prevent obesity or produce clinically meaningful weight changes.

The positive associations observed at higher chewiness levels may appear inconsistent with evidence linking prolonged chewing to slower eating, reduced food intake, and greater satiety [23,24]. However, these studies examined different exposures and outcomes. Experimental studies manipulated the number of chews before swallowing and assessed immediate eating behavior or appetite responses, whereas our study examined habitual intake of heterogeneous foods classified by estimated chewing requirements in relation to existing weight status. Consuming more food within a higher chewiness category does not necessarily indicate more chews per bite, longer oral exposure, or slower eating. Because these behaviors and satiety responses were not measured, the present findings cannot establish whether the mechanisms demonstrated experimentally operated in this population.

Several considerations may explain the apparent discrepancy. Foods within the higher chewiness categories differed in nutritional composition and degree of processing; consequently, their associations with weight status may reflect broader dietary patterns rather than chewing requirements alone. Adjustment for total reported energy intake does not eliminate these differences or potential dietary reporting errors. Moreover, an energy-adjusted association does not directly test the proposed pathway through which prolonged chewing might influence weight by reducing energy intake. Reverse causation is also possible, because existing weight status may influence food choices and reported consumption. Finally, short-term changes in eating rate, appetite, or meal intake do not establish sustained changes in body weight. These considerations offer plausible explanations, but the available data cannot determine which accounts for the positive association. Thus, our findings should not be interpreted as evidence that prolonged chewing increases obesity risk or that its potential effects on satiation are absent.

A few studies show that higher BMI is associated with a more potent bite force; others find no association [23,25,26]. Denture insertion has been shown to improve chewing function but not nutrient intake or weight outcomes [27]. Harder food texture may require an increased number of chews to break down the food and swallow it [28]. Experimental studies indicate that increasing chewing can slow eating and, in some settings, reduce meal intake; however, these short-term findings do not establish sustained benefits for body weight [23,29]. Potential physiological mechanisms involving digestion and appetite regulation are discussed below, although their contribution to the associations observed in the present study remains untested.

Previous research suggests that chewing behavior may influence appetite and food intake [23,24]. However, chewing behavior, satiety responses, and gastrointestinal hormones were not assessed in this study, and their contribution to the observed associations remains untested. Potential pathways include appetite regulation by modulating gut hormones such as Ghrelin, Glucagon-like peptide-1 (GLP-1), and Cholecystokinin (CCK), which affect hunger and satiety [24,30]. These mechanisms represent possible explanatory hypotheses for associations involving food texture. Their relevance to the present findings remains untested, as eating rate, chewing duration, number of chewing cycles, bite size, and satiety responses were not assessed.

Early and repeated exposure to varied textures fosters oral processing skills, healthier food acceptance, and self-regulation of intake [31]. Moreover, children who prefer softer textures may consume a less varied diet and have a higher risk of obesity due to overconsumption of energy-dense, easily consumed foods [32]. Harder foods require more chewing, which increases oral exposure and enhances expected satiation, often leading to smaller meal portions [9,33]. However, children living with overweight or obesity frequently display altered chewing patterns, including fewer mastication cycles and faster eating rates, which are associated with greater intake of energy-dense foods and lower consumption of fiber-rich foods, such as fresh vegetables and fruits [34,35]. Such behaviors may reflect specific sensory preferences, like favoring softer, easier-to-eat foods, potentially reinforcing obesogenic eating habits [5,36].

Sensory cues, including taste, texture, and palatability, activate reward pathways that influence both food choice and hedonic eating [33]. The integration of multiple sensory inputs, such as taste, smell, and texture, can sometimes override satiety signals, particularly in environments dominated by ultra-processed foods [33,37]. Interventions that promote sensory education, such as repeated exposure to a variety of textures and encouraging slower, more mindful eating, may help children better recognize fullness cues and reduce overall energy intake [38,39]. Whether modifying food texture or chewiness can improve weight outcomes requires evaluation in controlled intervention studies and cannot be inferred from the present observational findings.

This study has several limitations. The use of secondary data analysis restricted the ability to assess specific food textures comprehensively, and the chewiness classification was based on a post-hoc assessment by dietitians, potentially introducing misclassification bias. Self-reported dietary data may have been underreported, particularly among participants with overweight and obesity. Additionally, the lack of direct measurements of eating rate, chewing duration, number of chewing cycles, and bite size precludes determining whether these behaviors explain the observed associations. Furthermore, the cross-sectional design precludes causal inference, emphasizing the need for longitudinal studies to clarify temporal relationships, and does not establish whether dietary intake preceded participants’ current weight status. Reverse causation remains possible, as existing weight status may influence food choices, quantities consumed, attempts to modify the diet, and dietary reporting. These pathways were not directly assessed and remain potential explanations for the observed associations. Lastly, foods within each chewiness level differed in energy density, nutrient composition, degree of processing, and physical characteristics. For example, Level 4 included fresh fruits and vegetables, nuts, biscuits, and cereal bars. Consequently, the chewiness classification may function partly as a marker of food choices and broader dietary patterns, and the present analysis cannot distinguish associations attributable to chewiness from those related to food composition or food type. Adjustment for total energy intake does not fully account for these characteristics, and residual confounding remains possible. Additional adjustment for the degree of food processing was not feasible because the available FFQ data lacked sufficient detail for reliable classification. For example, bread items did not capture the product formulation or manufacturing methods needed to distinguish different levels of industrial processing.

Although the high inter-rater agreement supports consistency among the evaluators, it does not establish whether the assigned levels correspond to objectively measured differences in food properties or oral processing demands. Future validation should combine instrumental characterization of representative foods from all five levels with direct assessment of oral processing. Mechanical testing could quantify the hardness and fracture properties of solid foods, while rheological measurements could characterize liquids and semi-solids [40]. Standardized eating tasks could then assess the number of masticatory cycles per bite and oral-processing duration using video recordings [9]; surface electromyography could provide complementary information on masticatory muscle activity [41]. Such testing should include common preparation variants, control bite size, and serving conditions, and account for participants’ age and oral health. Examining ordered differences between levels, together with variability within levels, would help determine whether the proposed categories discriminate chewing requirements and whether particular foods require reclassification. Accordingly, these findings should be interpreted as associations with the consumption of foods grouped by estimated chewing requirements, rather than as evidence of an independent contribution of chewiness to overweight/obesity.

Among the strengths of this study are the large, nationally representative sample of adolescents and the availability of data on unhealthy behaviors, dietary intake assessed using a validated FFQ, and demographic characteristics. Data was collected anonymously using standardized and validated tools, including questionnaires with established internal construct validity, thereby minimizing the risk of response bias. Additionally, anthropometric measurements were performed by trained study personnel, reducing the potential for misclassification. Another strength is the use of SES classifications based on criteria from the Israeli Ministry of Education, which helped avoid inaccuracies associated with self-reported SES by adolescent participants.

Future longitudinal studies should examine whether intake of foods classified by estimated chewing requirements precedes changes in weight status. Controlled interventions are needed to test whether defined modifications to food texture alter oral processing, energy intake, or body weight. Additionally, the development of validated tools to assess texture-specific dietary intake in children and adolescents could provide more accurate estimates of chewiness-related consumption and its implications for energy balance. Further investigations should also consider the potential moderating effects of psychological stress, as previous studies have identified stress-induced changes in eating behavior that may exacerbate weight gain [42]. Integrating measures of stress, satiety, and texture perception could yield more comprehensive insights into the complex interplay between sensory food properties, emotional regulation, and the likelihood of living with overweight or obesity.

## 5. Conclusions

In this cross-sectional study of adolescents, higher reported intake of foods assigned to higher estimated chewiness levels was associated with greater odds of prevalent overweight/obesity after adjustment for measured covariates. These findings do not establish temporal direction or demonstrate that food chewiness or greater mastication causes obesity. Reverse causation and residual confounding remain possible. Objective validation of the classification and longitudinal and experimental studies are needed to clarify these associations.

## Figures and Tables

**Table 1:** Demographics, anthropometric, health-related behaviors, and nutritional characteristics of the study population by weight status.

Variable	All (*n* = 3428)	Normal Weight (*n* = 2330)	Overweight/Obesity (*n* = 1098)	*p*-Value
Demographics				
Age (years)	15.2 ± 1.6	15.3 ± 1.6	15.1 ± 1.6	0.001
Sex (% males)	1557 (47.7)	1029 (46.6)	528 (50.2)	0.046
Population group (%Jews)	2346 (78.2)	1635 (79.6)	711 (74.9)	0.002
Socioeconomic status (%low)	1190 (32.7)	787 (32.1)	403 (34.2)	0.22
School (%high school)	1501 (48.7)	1060 (50.5)	441 (45.0)	0.003
Anthropometrics				
Height Z-score	−0.059 ± 1.021	−0.085 ± 1.006	0.003 ± 1.048	0.03
BMI Z-score	0.494 ± 1.072	−0.067 ± 0.708	1.725 ± 0.574	<0.0001
Health-Related Behaviors				
Physical activity (% as recommended) [17]	1083 (34.5)	733 (33.9)	350 (35.9)	0.27
Sleep duration (% as recommended) [18]	1908 (56.9)	1303 (57.2)	605 (56.3)	0.60
Screen time (% as recommended)	570 (20.0)	397 (19.4)	173 (20.3)	0.55
Nutritional Intake				
Energy (kcal/d)	2274.1 ± 1024.6	2310.5 ± 1041.7	2194.2 ± 983.2	0.003
Total Protein (% Energy)	12.0 (10.6–13.6)	12.0 (10.5–13.4)	12.2 (10.7–14.1)	<0.0001
Total Carbohydrate (% Energy)	54.4 (50.3–58.6)	54.8 (50.8–59.0)	53.5 (49.1–58.0)	<0.0001
Total Fats (% Energy)	31.2 (28.2–33.9)	31.0 (28.1–33.8)	31.5 (28.3–34.3)	0.002
Food Intake According to Chewing Level Median (IQR)				
level 0 (grams/d)	737.0 (428.0–1194.4)	735.0 (413.6–1204.6)	737.1 (451.9–1175.9)	0.91
level 1 (grams/d)	149.7 (83.5–253.8)	150.7 (84.2–253.3)	145.5 (81.7–254.7)	0.43
level 2 (grams/d)	633.5 (442.4–898.6)	643.3 (449.0–918.4)	607.8 (423.7–860.8)	0.016
level 3 (grams/d)	398.3 (258.4–592.4)	398.3 (257.4–587.2)	398.3 (260.9–602.5)	0.54
level 4 (grams/d)	230.5 (115.9–404.6)	228.2 (114.9–395.3)	233.3 (119.7–418.7)	0.041

Calculated with the application of sample weights of the Israeli Youth Health and Nutrition Survey. Categorical variables are expressed as *n* (%) and continuous variables are expressed as mean ± SD or medians and IQR (25th–75th).

**Table 2:** Associations between food intake at each chewiness level and overweight/obesity in multivariable survey logistic regression models *.

	Outcome: Overweight/Obesity		Outcome: Overweight/Obesity OR (95% CI) per 10-g/Day Higher Reported Intake	
Food Intake by Chewiness Level, per 10-g/Day Increment	Model 1: OR (95% CI)	*p* -Value	Model 2: OR (95% CI)	*p* -Value
Level 0 (Lowest)	1.000 (0.999–1.001)	0.640	1.001 (1.000–1.002)	0.094
Level 1	0.997 (0.993–1.002)	0.241	1.003 (0.997–1.009)	0.284
Level 2	0.999 (0.997–1.001)	0.215	1.002 (1.000–1.005)	0.053
Level 3	1.001 (0.999–1.003)	0.428	1.007 (1.003–1.010)	<0.001
Level 4 (Highest)	1.004 (1.001–1.007)	0.019	1.006 (1.003–1.010)	<0.001

* Results of multivariable survey logistic regression models, adjusted for age, sex, socioeconomic status, and population group, with analyses accounting for the sampling design and survey weights. Model 1: Adjusted for age, sex, socioeconomic status, and population group. Model 2: Model 1 with additional adjustment for total energy intake. In Model 1, only food intake from the highest chewiness level (Level 4) (per 10-g/day increment) was significantly associated with overweight/obesity (*p* = 0.019). Following additional adjustment for total energy intake in Model 2, significant associations were observed for food intake from chewiness Levels 3 and 4.

## Data Availability

Requests for materials should be addressed to Tali Sinai.

## Supplementary Materials

The following supporting information can be downloaded at https://www.mdpi.com/article/10.3390/nu18213677/s1, Table S1. Assumptions for the central illustrative scenario. Table S2. Approximate required sample sizes under alternative assumptions [43,44]. Table S3: Fully adjusted survey-weighted logistic regression coefficients, school-bootstrap variance–covariance matrix, and linear trend across food chewiness levels.

## Abbreviations

The following abbreviations are used in this manuscript:

T2D	Type 2 diabetes
CVD	Cardiovascular disease
OECD	Organization for Economic Co-operation and Development
US	United States
SES	Socioeconomic status
GLP-1	Glucagon-like peptide-1
CCK	Cholecystokinin
BST	Behavioral Susceptibility Theory

## Appendix A

**Table A1:** Classification of Foods from the FFQ of Second MABAT Youth Survey by Chewing Level.

Food Item
Chewing Level 0
Cow’s milk (irrespective of fat %)
Chocolate drink (based on milk)
Olive or canola oil (not including oil in salad)
Sugar (added to drinks/cereals/yogurt, etc.)
Water
Carbonated soft drink (Coke, Sprite, etc.)
Diet carbonated soft drink (with no sugar) (Diet Coke, Diet Sprite, etc.)
Energy drink (XL or BLU)
Sweet soft drink such as Spring or water with syrup
Flavored water
Fruit juice drink/nectar (orange, apple, banana, strawberry, or other)
Coconut juice
Tea—any kind
Coffee (including with milk)
Chewing Level 1
Leben, unsweetened yogurt
Yogurt with Fruit or Other Topping (granola, chocolate, etc.)
Chocolate/vanilla pudding
Sour Cream
White cheese (including additional ingredients, e.g., olives) (A container of 250 g has 6 tablespoons of cheese)
Cottage cheese (including additional ingredients, e.g., olives) (A container of 250 g has 6 tablespoons of cheese)
Hummus salad
Tahini salad
Ful (broad bean) salad
Cream cake or cheese cake
Potatoes, mashed
Avocado (including avocado salad)
Margarine, butter
Mayonnaise, any type including salad dressing
Chocolate spread
Ketchup
Honey or jam
Ice cream (on stick)—dairy
Ice cream
Chewing Level 2
Yellow cheese
Salty cheese, e.g., Bulgarian cheese
Meatballs: beef, chicken, turkey
Sausages (hot dogs)
Tuna (in oil/water)
Egg, fried, omelet, scrambled, or “shakshuka”
Egg, soft/hard boiled
Falafel
White bread, Challah including toast
Bread, whole wheat or rye including toast
Light/diet bread (all types) including toast
White bun (roll)
Pita
Rice, cooked
Pasta, spaghetti, noodles, short-cut pasta
Couscous with or without gravy
Pizza
Plain cake, e.g., chocolate or marble cake
Individual pastry or Danish (e.g., croissant)
Banana
Watermelon, carambola (star fruit)
Other fruit, e.g., persimmon, mango, guava, strawberries, grapes
French fries
Potatoes, cooked or baked in the oven
Zucchini or eggplant baked or cooked
Other cooked vegetable, e.g., peas, mushrooms, pumpkin, spinach, etc.
Tomatoes including cherry tomato (including in a sandwich, not including in salad)
Vegetable soup
Salty snacks—e.g., crisps, Doritos (Tortilla chip crisp), Cheetos (cheese snack), Bamba (peanut butter flavored snack), pretzels
Popsicle
Injera (Ethiopian bread)
Tabun bread
Pita with Za’atar
Ptira sandwich (ethnic Arab bread)
Chewing Level 3
Beef, Steak, Roast
Chicken
Schnitzel (chicken/turkey)
Hamburger or kebab
Sausage or pastrami
Fish, fried or baked
Vegetarian schnitzel/sausage (including corn schnitzel)
Crackers, any kind
Unsweetened breakfast cereals, e.g., cornflakes or branflakes
Sweetened breakfast cereals, e.g., Frosties or Cocopops
Breakfast cereals with filling, e.g., Crunch
Burekas
Melawach, Jahnun
Citrus fruit, e.g., orange, tangerine
Plums, peaches, nectarines
Melon
Corn, Sweet corn
Green beans, cooked
Broccoli or Cauliflower
Cooked legumes, (e.g., chickpeas, white beans, lentils)
Cabbage salad
Cucumber (including in a sandwich, not including in salad)
Olives
Pickled vegetables, e.g., cucumber, eggplant, tomato, etc.
Chocolate (squares)
Chocolate bar—Mars Bar, Maltesers, Kit Kat, etc.
Candies—any kind
Chewing Level 4
Seeds (sunflower, pumpkin, watermelon)
Walnuts, almonds, pistachios, peanuts
Cookies or wafers
Apple, pear
Fresh vegetable salad
Peppers—all colors
Other fresh vegetables (e.g., kohlrabi, fennel)
Cereal snack, Energy bar (e.g., Shugi, Nature Valley)
