# Supplementary material for: Association Between Consumption of Foods Classified by Chewiness Level and Overweight/Obesity Among Israeli Adolescents: Insights from a Cross-Sectional National Youth Survey
Source: Nutrients. 2026 Nov 6;18(21):3677. doi: 10.3390/nu18213677 (PMC13765928; doi:10.3390/nu18213677)
Supplement: Supplementary file 1 [file nutrients-18-03677-s001.zip › nutrients-4622260-supplementary.pdf]

Supplementary Methods

Illustrative sample size calculations

Purpose and analytical context

The analytic sample was determined by the availability of eligible records from the MABAT Youth 2 Survey and comprised 3428 adolescents. The calculations below were undertaken during manuscript revision to illustrate how sample size requirements vary under the stated assumptions. They did not determine recruitment or sample selection. They are not estimates of achieved power based on the observed associations. For an analysis of existing data, the available sample and the precision of the reported estimates remain the primary considerations [43].

Calculation method and assumptions

For one continuous exposure coefficient in a logistic regression model, we used the approximation described by Hsieh et al. (1998), with an additional assumed design-effect multiplier:

$$n = \left\lceil \frac{D \times (z_{1-\alpha/2} + z_{1-\beta})^2}{p* \times (1 - p*) \times [ln(OR_{SD})]^2 \times (1 - R_X^2)} \right\rceil$$

Here, z denotes a standard normal quantile, $1 - \beta$ is statistical power, and the ceiling operator rounds the calculated sample size upward. Table S1 defines the remaining parameters.

Table S1. Assumptions for the central illustrative scenario

Parameter	Value	Rationale and interpretation
Two-sided α	0.05	Conventional significance level for a single coefficient test.
Power	80%	Conventional illustrative planning target.
Event probability p*	0.30	Assumed probability of overweight/obesity at mean exposure. Close to the unweighted sample proportion of 32.0% (1098/3428), but not an estimate of that conditional probability.
OR per one SD	1.20	Hypothetical modest association, selected independently of the observed ORs; not an established clinically important threshold.
Exposure R^2_X	0.25	Assumes other model covariates explain 25% of exposure variance. This is not a logistic pseudo-R^2 and was not estimated from the data.
Design effect D	1.50	Assumed 50% variance inflation for the exposure coefficient under the survey design; not an empirical design-effect estimate.
Exposure distribution	Continuous	Approximately normal for this calculation; robustness to skewed FFQ intakes has not been assessed.

Worked example and alternative scenarios

Using $z_{0.975}$ = 1.959963985 and $z_{0.80}$ = 0.841621234, the central scenario gives:

$$n = \text{ceiling}\{1.5 \times (1.959963985 + 0.841621234)^2 / [0.30 \times 0.70 \times \ln(1.20)^2 \times 0.75]\} = \text{ceiling}(2248.756) = 2249.$$

Calculations used full-precision standard normal quantiles and were rounded upward only at the final step. Table S2 varies the hypothesized association and assumptions about covariate correlation and the sampling design; α, power, and p* remain unchanged.

Table S2. Approximate required sample sizes under alternative assumptions [43, 44]

OR per one SD	Exposure R^2_X	Design effect	Required sample
1.15	0.25	1.5	3827
1.20	0.25	1.5	2249
1.25	0.25	1.5	1502
1.20	0.50	2.0	4498

Note. OR = odds ratio; SD = standard deviation. Each row applies to one exposure coefficient. No allowance for missing data is added because the comparison is with the final analytic sample, after exclusions.

Interpretation and scope

The available sample of 3428 adolescents exceeds the estimated requirements for ORs of 1.20 and 1.25 when R^2_X = 0.25 and D = 1.5. It does not exceed the requirement for OR = 1.15 under those assumptions, or for OR = 1.20 when R^2_X = 0.50 and D = 2.0. Thus, the available sample meets the requirements of some illustrative scenarios, but does not establish adequate power for every plausible association or model.

The ORs in these calculations refer to a one-SD increase in intake; the manuscript reports ORs per 10 g/day. Conversion requires the SD of intake within the relevant chewiness level: $OR_{SD} = (OR_{10g})^{\frac{SD_g}{10}}$. The scenarios are not calibrated to the observed exposure distributions, covariate correlations, or survey-design effects. They concern a single coefficient test at α = .05, without adjustment for multiple testing, and do not establish power for all five chewiness levels, subgroup analyses, or the Joinpoint analysis. Precision should be judged using confidence intervals from models that appropriately account for the sampling design.

Supplementary Table S3
Chewiness coefficients and linear trend

Survey-weighted logistic regression coefficients, school-bootstrap variance–covariance matrix, and linear trend across food chewiness levels.

Logistic regression coefficients

Chewiness level	Log-odds coefficient	Taylor standard error	Bootstrap standard error	p value
0	0.00093193	0.00055308	0.00055463	0.0935
1	0.00303676	0.00282396	0.00281262	0.2835
2	0.00232999	0.00119803	0.00125887	0.0531
3	0.00655553	0.00176055	0.00180327	0.0003
4	0.00647680	0.00169282	0.00170399	0.0002

B Bootstrap covariance matrix

Displayed entries equal the covariance multiplied by 10^6.

Level	0	1	2	3	4
0	0.30761491	−0.17838892	−0.12404755	−0.11002405	−0.03726450
1	−0.17838892	7.91082570	0.38082743	0.08763490	0.54288290
2	−0.12404755	0.38082743	1.58474110	0.15214698	0.48670876
3	−0.11002405	0.08763490	0.15214698	3.25177610	0.90335807
4	−0.03726450	0.54288290	0.48670876	0.90335807	2.90358730

C Linear trend

Trend slope	Standard error	95% confidence interval	df	p value
0.00137455	0.00041657	0.00055328–0.00219581	207	0.0011

The analyses included 3428 participants in 208 schools. The outcome was prevalent overweight/obesity. Each coefficient in panel A represents the log-odds ratio per 10 g/day increase in intake within the indicated chewiness level. Five separate models were adjusted for age, sex, socioeconomic status, population group, and total daily energy intake, with national survey weights and clustering at the school level. The p-values in panel A use Taylor standard errors and t-tests with 207 degrees of freedom.

Bootstrap standard errors and covariances were estimated from 2000 joint Rao–Wu rescaled samples. In each sample, 207 schools were drawn with replacement from the 208 schools; all their participants were retained; and survey weights were multiplied by the school selection multiplicity and by 208/207. All five models were refitted within each sample; all 2000 replicates were retained. The diagonal of panel B contains variances, and the off-diagonal entries contain covariances. Multiply each displayed entry by 10^{-6} to recover its original value.

Panel C presents a linear regression of the five log odds ratios on level-coded 0–4, with the full covariance matrix incorporated into the estimation of the regression coefficients and their standard errors. The slope represents the change in the log-odds ratio per one-unit increase in chewiness. Its confidence interval and two-sided p-value are based on an approximate Wald t-test with 207 degrees of freedom. Calculations used the unrounded SAS estimates. df, degrees of freedom.
